# Amplified Spontaneous Emission in Paper

**DOI:** 10.1038/s41598-018-38438-x

**Published:** 2019-02-12

**Authors:** N. M. Hoinka, T. Fuhrmann-Lieker

**Affiliations:** Macromolecular Chemistry and Molecular Materials, Institute of Chemistry and Center for Interdisciplinary Nanostructure Science and Technology, University of Kassel, Heinrich-Plett Str. 40, 34132 Kassel, Germany

## Abstract

Industrially produced copy paper is presented as a novel optical material for narrow-band stimulated emission. Fluorescent brightening agents (FBA) such as Calcofluor White provide enough gain to exceed the threshold for amplified spontaneous emission. By applying an additional dye such as Coumarin 307, or simply a highlighter pen, the emission line can be shifted from blue (~440 nm), towards turquoise (>480 nm), up to orange (590 nm) which can be useful for spectroscopic applications. These properties are demonstrated in two papers, a commercial copy paper and a FBA-stained calligraphy paper.

## Introduction

The construction of narrow-bandwidth emitting organic lasers relies on the accuracy of technical equipment, such as deposition techniques for the construction of optical waveguides and optical techniques for writing periodic resonant structures^1^. As an alternative approach, the concept of random lasers in which optical modes are guided and supplied with feedback within a disordered medium has been elaborated^2–4^. A wide range of gain materials have been developed, which are normally prepared on flat or structured substrates^5,6^. In the past decade, however, there has been an increased interest in simple material systems that are inexpensive, easy to build, flexible, and do not require many preparation steps to be operational. Recently, paper has emerged as a promising candidate to fulfil these requirements^7^. As a renewable resource, paper is an economically accessible, abundant and biodegradable composite that is already used in a variety of industrial applications (including, but not limited to copy paper, filters and weighting papers). Consisting of interconnected cellulose fibres often filled with mineral fillers, such as calcium carbonate, this composite material achieves better stability and smoothness as well as opaqueness compared to just cellulose fibres^8^. In order to improve the brightness and to maintain a white impression to the human eye, fluorescent brightening agents (FBA) are applied during the manufacturing process and attach onto the cellulose fibres. These molecules absorb within the UV region and possess a spectrally broad fluorescence within the visible blue region.

The potential of paper and cellulose for functional devices that exceed traditional applications has been explored recently. First, electroactive paper, i.e. paper as energy storage device and paper electronics represent promising electronic fields of application^8–10^. Furthermore, cellulose fibres, as a base for optically emissive structures, have recently gained traction. Random cellulose fibre systems in chromatographic paper can act as scattering centres when a dye solution is applied. In utilizing soft lithographic confinement in microfluidic channels, a net transition from non-resonant to resonant random lasing was demonstrated^11^. Finally, laser emission in transparent wood, a novel cellulose-based material, different to paper, was demonstrated. with the same rhodamine dye as in ref.^11^ used as an active medium^12^.

In this study, it is demonstrated that FBAs present in common, commercially used paper are sufficient for achieving narrow-band stimulated emission with spectral linewidths lower than 3 nm. Through the addition of a second solid-state organic laser dye such as Coumarin 307, or by using dye containing highlighter pens, the emission line can be shifted from blue (440 nm) up to orange (590 nm) with similar linewidths. With this property, paper-based light source arrays for spectroscopic sensor applications may be feasible, which fit in already established manufacturing and recycling processes.

Two different types of paper were analysed. The first paper used is “Xero 80 premium”, produced by Stora Enso Paper AB, Nymölla Mill in Sweden. It is a white standard copy paper containing inorganic filling material as well as an optical brightener not further described by the manufacturer. This paper was chosen as representative for the class of daily-use papers (paper A). The second paper, purchased from Wenzhou Haili Industry & Trade Co. in China does not contain any filling material, or optical brighteners and is normally used for calligraphy (paper B). Both papers have a fibre length in the range of several millimetres (see Supplementary Fig. S1), but differ in cellulose fibre density as well as thickness. In paper A, the space between fibres is covered with a filling material, whereas paper B, has free space between the fibres.

Since paper A already contains optical brighteners and shows enhanced scattering due to the filling material, it was used without further preparatory steps. In the case of paper B, it was either used without treatment (blind experiment) or the sheets were soaked in a solution of Calcofluor White (Fluorescent Brightener 28, Sigma-Aldrich) in demineralized water (1 mg/ml) and dried. This optical brightener is known to bind to cellulose, chitin, or polyamide, and is often used to colorize cell walls for fluorescence microscopy in biological experiments^13,14^. The respective spectra for both stained papers are shown in Supplementary Fig. S2. In order to measure the influence of an additional dye emitting at longer wavelength, a solution of 15 mg/ml of Coumarin 307 (Radiant Dyes) in CHCl_3_ (Uvasol®) was added drop-wise onto the two papers until they were soaked with the solution. Once the solvent evaporated, this step was repeated one more time. Chloroform was chosen because the FBAs used here are not well soluble in this solvent. Out of many Coumarin dyes, Coumarin 307 showed a good affinity towards cellulose fibres. This might be attributed to the secondary amine moiety interacting with negatively charged cellulose fibres. Furthermore, Pelikan Textmarker 490 highlighter pens in four different colours (green, yellow, orange and red) were used to stain the respective papers. Further information and spectra about the used dyes and mixtures can be found in the Supplementary Figures S3, S4 and Table S1.

For optical pumping, a LTB MSG 800 nitrogen laser with a wavelength of *λ* = 337.1 nm and a pulse duration less than 500 ps was either used with a continuous pulse frequency of 10 Hz or by triggering single pulses. The intensity was varied though a continuously adjustable neutral density filter and the beam was focused on an area of 2.63 mm². The emitted light scattered from the samples was collected at an angle of 45° and analysed by a detector array spectrometer (AvaSpec 3648) with a resolution of 0.2 nm. Emission and excitation spectra were carried out with a Hitachi F-4500 fluorescence spectrometer, and fluorescence life time experiments were performed with a FluoTime 200 (PicoQuant) life time measurement setup.

**Figure 1:**
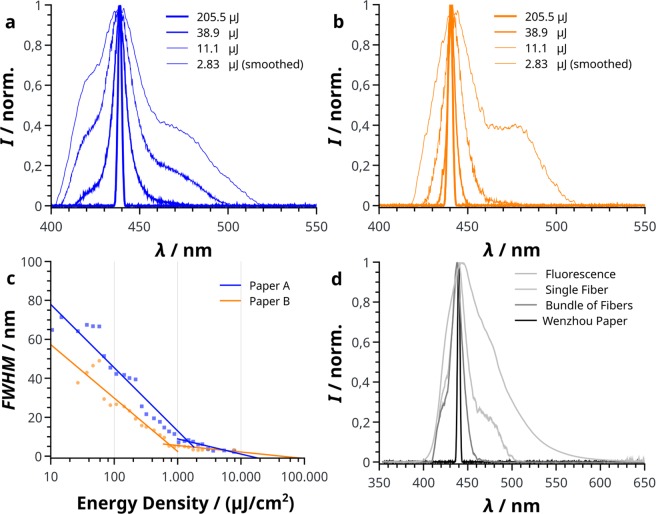
Emission spectra and threshold determination. Normalized emission spectra of (**a**) copy paper (blue) and (**b**) calligraphic paper stained with Calcofluor White (orange) at given input energy. It is clearly visible, that the FWHM decreases with increasing input intensity. For clarity the spectra at 2.83 µJ were smoothed with a Savitzky-Golay filter using a third order polynomial and taking a total of 20 points into account. (**c**) Shows the full width at half maximum at given energy density for paper A and B, respectively. The inflection point of the logistic fit function (see supplementary information) represents the ASE threshold. (**d**) From the outside to the inside: Fluorescence spectrum of paper B stained with Calcofluor White, emission spectra for a single fibre, a bundle of fibres taken from a stained calligraphic paper as well as the paper itself.

The emission properties for both paper types with their respective dyes are shown in Fig. 1(a,b), respectively. Unlike the FBA containing paper, unstained paper B does not show fluorescence when excited with a nitrogen laser pulse. With increasing input intensity, the full width at half maximum (FWHM) of the stained papers decreases from values near the range of the measured fluorescence emission (69 nm and 62 nm for untreated paper A and B stained with Calcofluor White, respectively) to typical amplified spontaneous emission (ASE) values with a FWHM as low as 2.3 nm and 3.3 nm, respectively. This change from a broad fluorescence spectrum to a single line with increasing input intensity is common for laser dye containing well confined waveguides when the excitation intensity exceeds the threshold intensity for ASE^15^ and show strong similarities in behaviour. ASE by stimulated emission occurs in a gain material in which the pumping area is sufficiently large. It is characterized by the absence of standing longitudinal modes by a laser resonator, however, a mode confinement is usually present in the form of a waveguide structure (“travelling wave lasing”) in thin films^16,17^. Here, despite the structural irregularity, the optical modes in the stained fibres appear to be extended enough to overcome the threshold for stimulated emission. Thus, the condition of the pump length being larger than the gain length is fulfilled. Since paper represents an unstructured (composite) material it is important to mention, that no localized, but travelling, weak scattering optical modes with respect to the diffusive constants are present in these amplified disordered systems^18^.

When a spot is irradiated with more than one pulse at higher intensities, degradation is observed. By changing the irradiated spot, the spectrum remains stable and the location of the maximum intensity varies within a range of 0.5 nm. However, a fluctuating value of maximum intensity was observed. Therefore, the classical plot of output intensity vs. input intensity is not favourable in order to determine the ASE threshold values. A shift of the sample and hence the illuminated area for a following pulse is possible but introduces an additional stochastic error due to the intrinsic inhomogeneity of the material. Thus, ref.^15^ in plotting the FWHM against pump intensity is followed. Figure 1(c) shows a sigmoidal behaviour, when the FWHM is plotted against the logarithmic energy density. Each value represents an average width out of at least three measured spectra. The threshold was determined by a logistic fit function shown in the supplementary materials and the inflection point represents the ASE threshold. The threshold energy density for the ASE in case of paper A is (137 ± 10) µJ/cm^2^, while paper B shows a threshold energy density of (88 ± 7) µJ/cm^2^. The difference in threshold may have many causes. The structure, density, the scattering behaviour (see Supplementary Fig. S1) and the staining density show substantial differences. With wavelengths of 439 nm for paper A and 440 nm for paper B stained with Calcofluor White, the maxima of the ASE peaks are very close to each other and indicate that both FBAs belong to the same chemical class. However, no exact information about the FBA used in paper A is available.

Both threshold values are significantly lower than the liquid Rhodamine system in ref.^11^ (500 µJ/cm^2^) and the linewidths reach lower values in these present all-solid samples. Furthermore, it should be mentioned that this effect is not limited to paper A or B and were detected with a multiple of commercially available copy papers in Europe. Ten different papers have been tested and a notable exception is recycled paper, in which the FBA concentration is too low for ASE to be detected.

**Figure 2:**
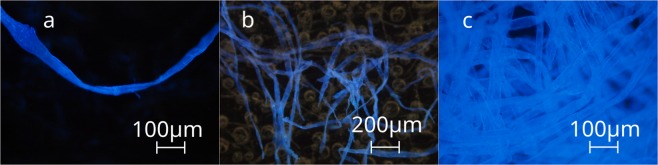
Fluorescence microscopic images of the analysed cellulose fibre (**a**), a bundle of fibres. (**b**) The tape can be seen in the background. Paper B is depicted in (**c**). The emission spectra are presented in Fig. 1(d).

Since the analysed systems exhibit a higher complexity compared to a simple slab waveguide the question remains whether a single fibre constitutes the complete optical path or coupling between several fibres is necessary to give optical feedback. Thus, a single fibre, fibre bundles and the interpenetrating network for their contribution to ASE (Fig. 2) are compared. Due to the presence of a coating layer as well as filling material on paper A, the fibres constituting paper B stained with Calcofluor White were chosen as a model and isolated using double-sided tape. From the attached bundle a single fibre was transferred to a second tape. The double-sided tape does not have any influence on optical measurements at the excitation wavelength. As can be seen in Fig. 1(d) a single fibre (shown in Fig. 2(a)) is sufficient to amplify the emission of a transition with a maximum wavelength at *λ* = 439 nm. By increasing the number of fibres, the spectrum slightly narrows from a FWHM of 21.5 nm down to 15.1 nm, with both values lower than the FWHM of the fluorescence measured for paper B with Calcofluor White (61.6 nm). The illuminated spot of the laser pulse has a larger diameter than a single fibre. Thus, the total area in which optical gain occurs is increased when a bundle is used. If the fibres acted independently, only an increase in intensity but no significant change in the FWHM would be visible. The observed decrease in FWHM from a single fibre to a bundle of fibres (Fig. 1(d)) suggests that the interconnected fibres create a coupled network, increasing the travel length and thus the intensity at the wavelength with maximum gain^19^.

Dyed single fibres are known for their ability to establish whispering gallery modes, a resonant mode around the perimeter of a fibre^3,20,21^. In case of the analysed fibre of paper B, no such modal spectra were observed. This can be attributed to the presence of an inhomogeneous diameter, hence preventing the manifestation of such resonant modes.

As a next step, with the aim of shifting the emission peak to higher wavelengths, the samples prepared with Coumarin 307 were analysed. Here, paper A as well as paper B (with and without Calcofluor White treatment) had to be considered. The overlap of the emission spectrum of the FBAs and the excitation spectrum of Coumarin 307 (see Supplementary Fig. S3(a) for Calcofluor White) allows the presence of, or at least the assisted wavelength shift by a Förster-type resonance energy transfer (FRET) system^22^.

In order to investigate whether a Förster-type resonance is present, the fluorescence lifetimes of paper B (Calcofluor White on FBA free paper) with and without the presence of Coumarin 307 are compared at a wavelength of 420 nm. For FBA stained paper B without Coumarin 307, a monoexponential reconvolution fit with a fluorescence lifetime of *τ* = (0.911 ± 0.004) ns and an amplitude of *A* = (15420 ± 100) counts was fitted with χ^2^(red) = 1.14 (see Supplementary Fig. S3(b)). An additional second exponential term did not improve the fit. On the other side, when Coumarin 307 is present, two exponential functions had to be used to obtain a good fit (χ^2^(red) = 1.07) for the experimental data. *τ*_1_ increased slightly to (0.967 ± 0.004) ns with and amplitude of (11800 ± 81) counts, while the second term showed a much shorter life time of *τ*_2_ = (0.36 ± 0.01) ns and a significant lower amplitude of (6550 ± 200) counts resulting in a mean lifetime of <*τ>* = (0.75 ± 0.02) ns. Considering that these experiments were carried out in solid state in which relaxation times may be broadened, a second exponential term for the fitting curve can indicate the presence of an alternative decay mechanism as the mean lifetime indicates, e.g. FRET, but does not stand as a sufficient criterion alone.

**Figure 3:**
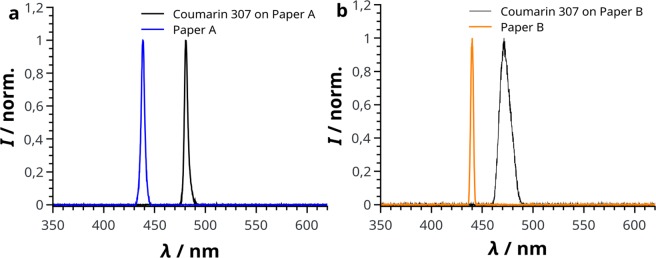
ASE emission spectra of the original paper A (blue) and B (purple) with only the corresponding FBA as well as colourized with Coumarin 307 (black lines).

Figure 3(a) shows both, the ASE spectrum of paper A without (blue line) and with Coumarin 307 (black line) with a redshift of 42.5 nm between the maxima and a FWHM of 3.5 nm, hence above the threshold for ASE. It is interesting to note that due to the degradation after the first pulse no further amplified spectrum (neither Coumarin 307 or FBA) can be detected at a previously irradiated spot. In contrast, the investigated papers with either Coumarin 307 or FBA, allow the reuse of the same excitation spot for a limited amount of pulses. Due to this fast degradation process, a determination of an ASE threshold for paper A (or B) containing both FBAs and Coumarin 307 was not possible. The addition of Coumarin 307 to the FBA-free calligraphic paper directly results in ASE although with a strongly reduced emitted intensity compared to paper A. This can be attributed to the thin and light structure of calligraphic paper. In combination with stained paper B no single pulse spectrum can be measured. However, the fluorescent spectra of Calcofluor White and Coumarin 307 are detected when the integration time and thus the number of exciting laser pulses is increased. To explore whether this may be attributed to reduced scattering in the illuminated area, CaCO_3_ was added to a solution of Calcofluor White, in order to fill the free space between the interpenetrating fibres and increase the scattering of light with the aim of higher local energy densities in the illuminated area surrounding the wave-guiding fibres. For a system of paper B stained with Calcofluor White and CaCO_3_, the threshold does not change. On the other hand, by further staining with Coumarin 307 an ASE peak was obtained, although with a far larger FWHM (11 nm) compared to the case of paper A stained with Coumarin 307 (Fig. 3(b)).

**Figure 4:**
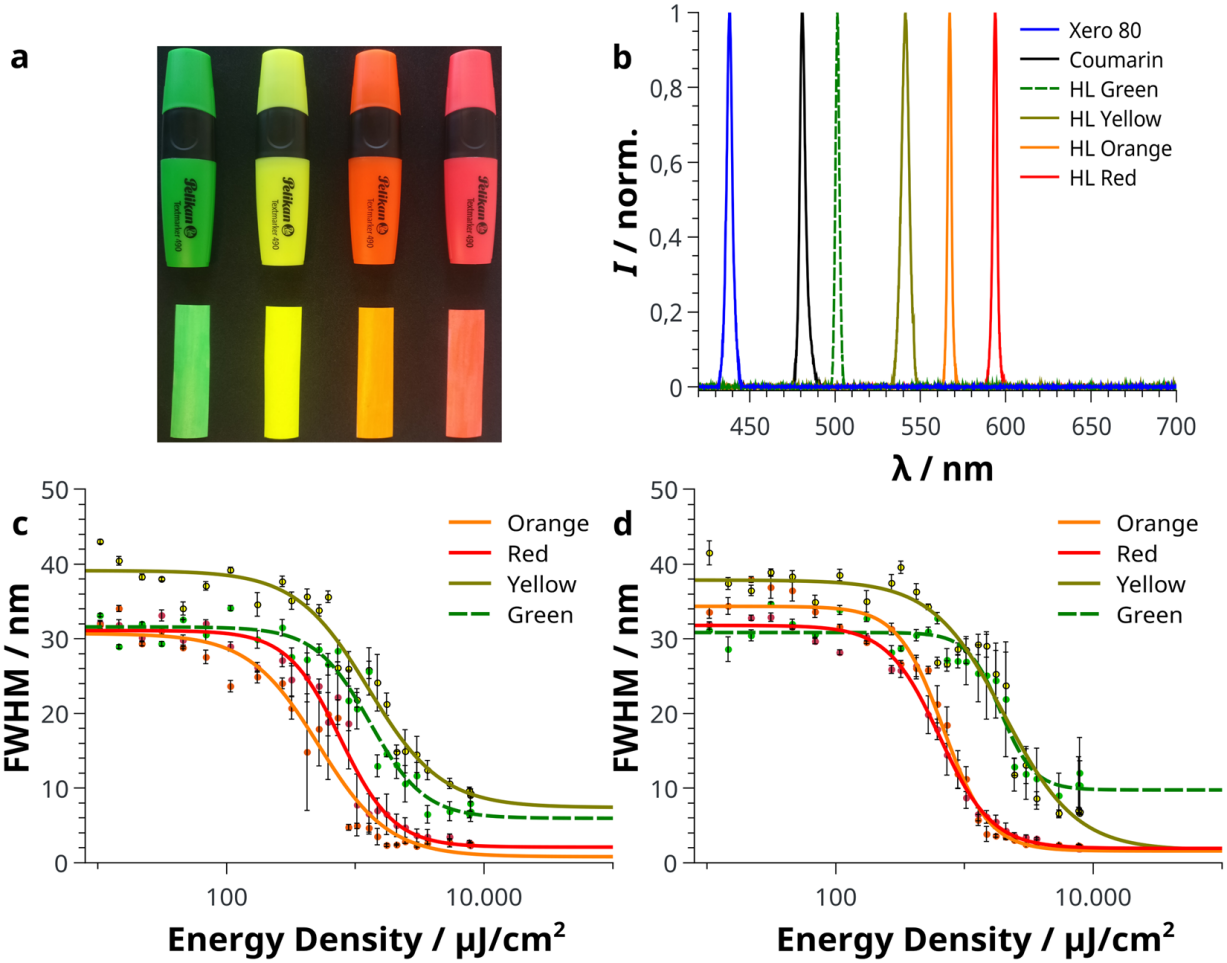
(**a**) Used highlighter pens as well as ready to use paper A samples. The resulting ASE spectra as well as the emission from paper A and paper A stained with Coumarin 307 are shown in (**b**). (**c**,**d**) The graphs show the FWHM at given (logarithmic) energy densities for threshold determination of highlighter dyes on paper A and paper B, respectively.

For Calcofluor White, as well as Coumarin 307, a good attachment onto cellulose fibres seems to be essential for amplified spontaneous emission. Thus, colourful highlighter pens appear to be a suitable material as they are meant to interact with cellulose and contain fluorescent dyes. The pens were used as delivered without further treatment. The application is not limited to samples treated with the Pelikan highlighters shown in Fig. 4(a) and can be transferred to other brands. Still, these highlighter pens stood out with high intensities and rather narrow ASE signals when analysed. The usage of commercially available dyes is a cost-efficient approach compared to available laser dyes. The highlighters consist of an aqueous mixture of dye molecules combined with acrylic esters, glycerol and additional preservatives^23^. The respective spectra, regardless of the paper type, appear to be stable when excited by a nitrogen laser pulse. Figure 4(c,d) show the spectral FWHM of the highlighter dyes at a given energy density for paper A and B, respectively. Like in Fig. 1, the FWHM decreases with increasing energy densities and thresholds in the range of 600 µJ/cm^2^ to 2000 µJ/cm^2^ are found by sigmoidal fitting of the data to obtain the inflection point. For a detailed threshold overview see Supplementary Table S1. Especially the orange and red highlighter appear to have reached saturation as the FWHM is in the range of 3 nm and stable. Figure 4(b) shows the broad spectral range from 438 nm to 590 nm of the performed ASE experiments on paper A and thus the full potential of this cost-efficient materials for optical applications. Possible future application of this technology could consist of a narrow band emitting flexible device composed of a small pulsed ultraviolet LED for optical pumping with an exchangeable roll of paper and a respective dye to, for example, emit at an analytical relevant wavelength in the visible range^24,25^. On the other hand, the usage of Coumarin 307 may find interesting applications as optical security system as the transition from ASE to fluorescence results in a single read-out and can also be used for destroying information encoded in the paper.

In conclusion, optically pumped narrow-band emission with FWHM as low as 2.3 nm in the blue spectral region can be achieved with simple copy paper containing fluorescent brightening agents. To the best of our knowledge, this is the first report for Calcofluor White as a material capable of lasing. By either incorporating known laser dyes such as Coumarin 307 or simply applying a highlighter, the ASE peak wavelength can be shifted from 439 nm up to 590 nm. The observed degradation of ASE after one or several pulses is not a serious issue in a mass-product such as paper in which a lateral shift from a paper roll may easily replace the active zone.

## Supplementary information

Supplementary Info

## References

[1] Kogelnik H, Shank CV (1971). Stimulated Emission in a Periodic Structure. Appl. Phys. Lett..

[2] Cao H (2003). Lasing in random media. Waves in Random Media.

[3] Polson RC, Chipouline A, Vardeny ZV (2001). Random Lasing in π-Conjugated Films and Infiltrated Opals. Advanced Materials.

[4] Wiersma DS (2013). Disordered photonics. Nature Photonics.

[5] Träger, F. (Ed.) *Springer Handbook of Lasers and Optics*. (Springer New York, 2007).

[6] Kuehne AJC, Gather MC (2016). Organic Lasers: Recent Developments on Materials, Device Geometries, and Fabrication Techniques. Chem. Rev..

[7] Nongbe MC (2018). Cellulose paper grafted with polyamines as powerful adsorbent for heavy metals. Cellulose.

[8] Tobjörk D, Österbacka R (2011). Paper Electronics. Adv. Mater..

[9] Khan A, Abas Z, Kim HS, Kim J (2016). Recent Progress on Cellulose-Based Electro-Active Paper, Its Hybrid Nanocomposites and Applications. Sensors.

[10] Yao B (2017). Paper-Based Electrodes for Flexible Energy Storage Devices. Advanced Science.

[11] Ghofraniha N (2013). Transition from nonresonant to resonant random lasers by the geometrical confinement of disorder. Opt. Lett., OL.

[12] Li Y, Vasileva E, Sychugov I, Popov S, Berglund L (2018). Optically Transparent Wood: Recent Progress, Opportunities, and Challenges. Advanced Optical Materials.

[13] Herth W, Schnepf E (1980). The fluorochrome, calcofluor white, binds oriented to structural polysaccharide fibrils. Protoplasma.

[14] Zaas AK (2008). Plasminogen Alleles Influence Susceptibility to Invasive Aspergillosis. Plos Genetics.

[15] Salbeck J, Schörner M, Fuhrmann T (2002). Optical amplification in spiro-type molecular glasses. Thin Solid Films.

[16] Chénais Sébastien & Forget Sébastien (2012). Recent advances in solid-state organic lasers. Polymer International.

[17] Saragi TPI, Spehr T, Siebert A, Fuhrmann-Lieker T, Salbeck J (2007). Spiro Compounds for Organic Optoelectronics. Chem. Rev..

[18] Sheng, P. *Introduction to Wave Scattering*, *Localization and Mesoscopic Phenomena*. **88**, (Springer Berlin Heidelberg, 1995).

[19] Shaklee KL, Leheny RF (1971). Direct determination of optical gain in semiconductor crystals. Appl. Phys. Lett..

[20] Samuel IDW, Turnbull GA (2007). Organic Semiconductor Lasers. Chem. Rev..

[21] Polson RC, Raikh ME, Vardeny ZV (2002). Random lasing from weakly scattering media; spectrum universality in DOO–PPV polymer films. Physica E: Low-dimensional Systems and Nanostructures.

[22] Berggren M, Dodabalapur A, Slusher RE, Bao Z (1997). Light amplification in organic thin films using cascade energy transfer. Nature.

[23] Textmarker 490/414, Safety data sheet in accordance with 1907/2006/EC, https://extranet.pelikan.com/extranet/Pulsar/en_US.Extranet.displayExtranet.19233./textmarker-490-414-english (last modified, 15/1/2014).

[24] Riedl T (2006). Tunable organic thin-film laser pumped by an inorganic violet diode laser. Appl. Phys. Lett..

[25] Schneider D (2005). An Ultraviolet Organic Thin-Film Solid-State Laser for Biomarker Applications. Advanced Materials.

