# Supplementary material for: Amplified Spontaneous Emission in Paper
Source: Sci Rep. 2019 Feb 12;9:1862. doi: 10.1038/s41598-018-38438-x (PMC6372576; doi:10.1038/s41598-018-38438-x)
Supplement: Supplementary file 1 — Supplementary Info [file 41598_2018_38438_MOESM1_ESM.docx]

**Amplified Spontaneous Emission in Paper**

**Supplementary Information**

N. M. Hoinka^1)^, T. Fuhrmann-Lieker^1,*^

^1^ *Macromolecular Chemistry and Molecular Materials, Center for Interdisciplinary Nanostructure Science and Technology, University of Kassel, Heinrich-Plett Str. 40,*

*34132 Kassel, Germany*

**^*^ *Author to whom correspondence should be addressed. Electronic mail: th.fuhrmann@uni-kassel.de***


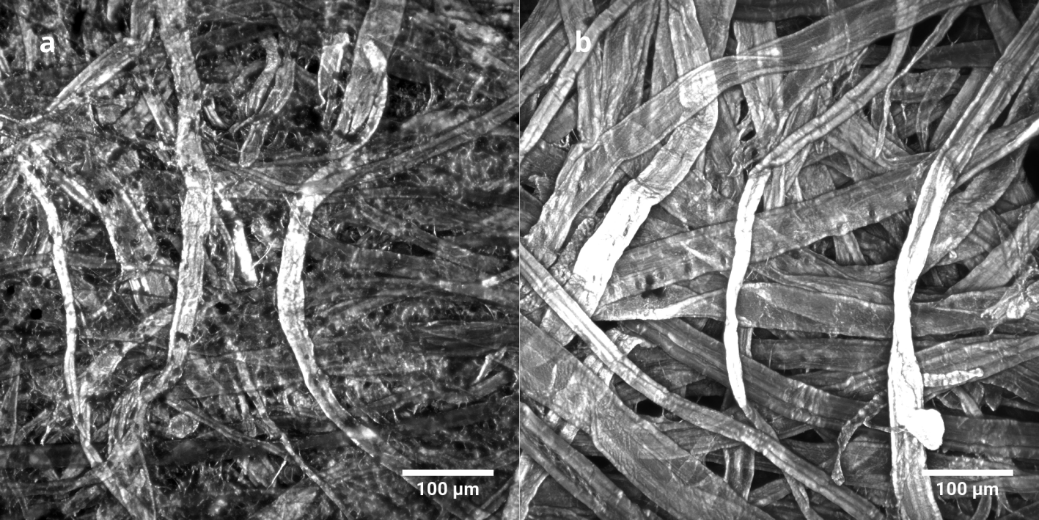


**Figure S1.** CLSM microscopic measurements as a z-projection of paper A (**a**) and paper B (**b**), respectively. The black shades spots in (**a**) are non-luminescent mineral fillers, while (b) does not contain any.


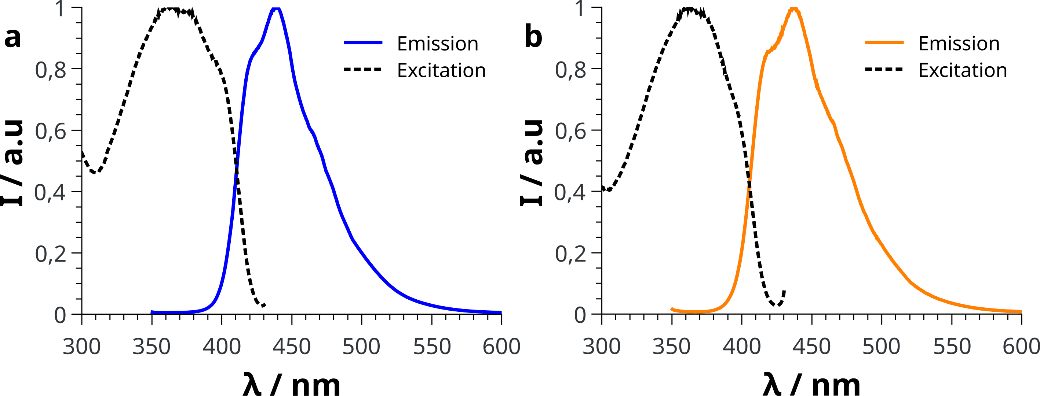


**Figure S2.** Excitation and emission spectra of (**a**) copy paper and (**b**) calligraphic paper stained with Calcofluor White.


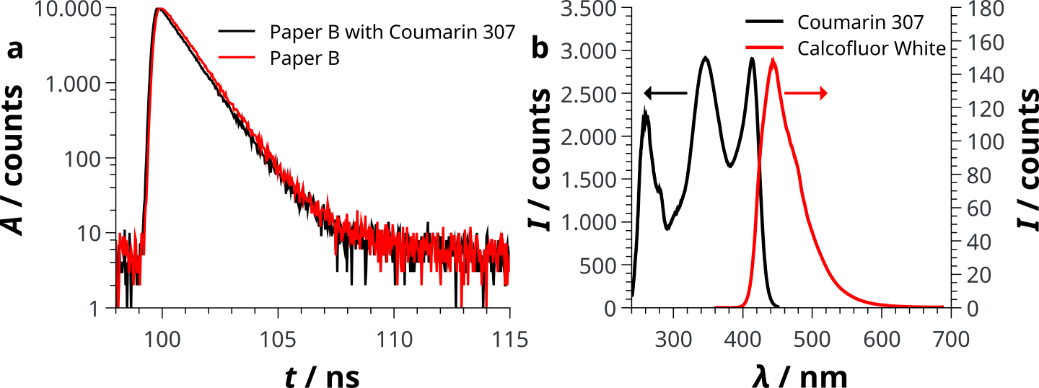


**Figure S3.** Solid state fluorescent lifetime measurement (**a**) at $\lambda=420$ nm of paper B (Wenzhou paper with Calcofluor White) with and without Coumarin 307 added. The overlap of the excitation spectrum of Coumarin 307 and the emission spectrum of Calcofluor White, both on paper B is shown in (**b**).


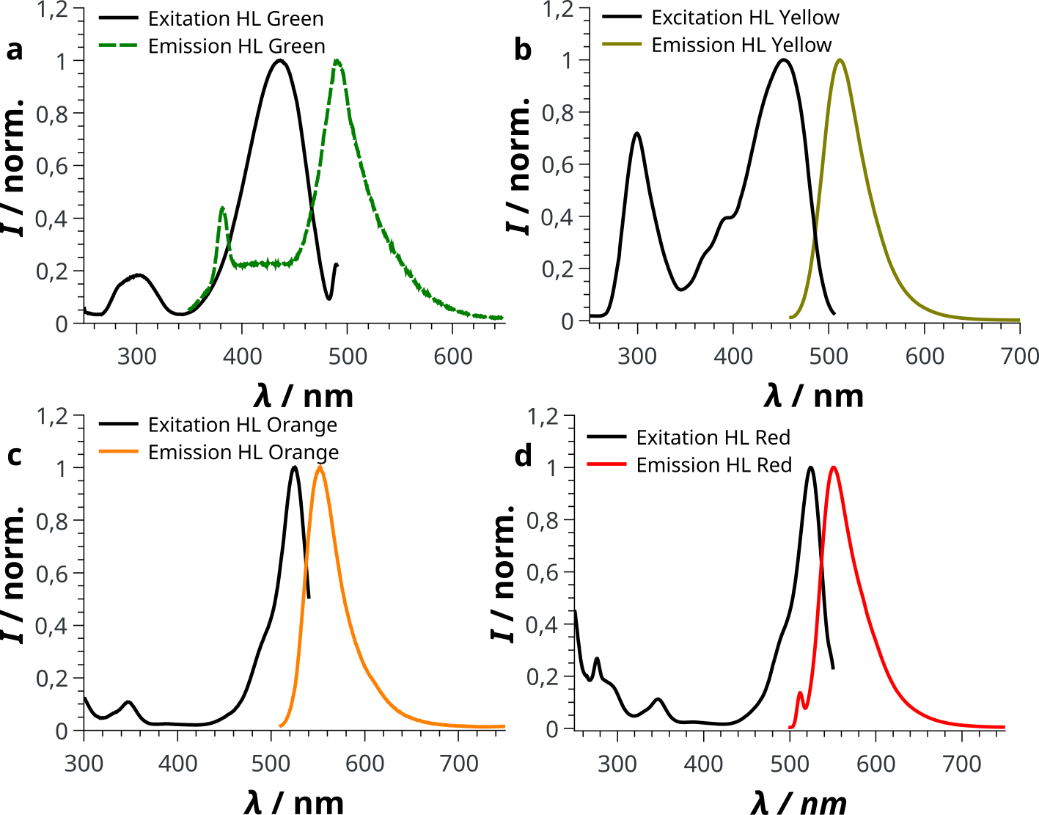


**Figure S4.** Excitation and emission spectra of the analysed highlighter dye mixes (green (**a**), yellow (**b**), orange (**c**) and red (**d**)) measured in distilled water.

**Table S1.** Threshold obtained from Fig. 4 (a) and (b) using a sigmoidal fit. x_0_ represents the turning point and is defined as threshold.

| **Highlighter** | **Paper A** | | **Paper B** | | |
| --- | --- | --- | --- | --- | --- |
|  | *x*_0_ /µJ/cm^2^ | *Δ x*_0_ / µJ/cm^2^ | | x_0_ / µJ/cm^2^ | Δ x_0_ / µJ/cm^2^ |
| **Green** | 1300 | 140 | | 1900 | 150 |
| **Yellow** | 1272 | 180 | | 2045 | 425 |
| **Orange** | 520 | 75 | | 698 | 40 |
| **Red** | 768 | 65 | | 649 | 30 |

A logistic function of the type

$FWHM\left( x \right)=A_{2}+\frac{A_{1}-A_{2}}{1+\left( \frac{x}{x_{0}} \right)^{p}}$

with *x* as fluence used for fitting. *A*_1_ and *A*_2_ describe the respective FWHM at low and high values for *x*. *x*_0_ is the inflection point representing the threshold and *p* is the steepness of the function.
